# Identification of genes associated with laryngeal squamous cell carcinoma samples based on bioinformatic analysis

**DOI:** 10.3892/mmr.2015.3794

**Published:** 2015-05-18

**Authors:** BO YANG, XUELI BAO

**Affiliations:** Department of Otolaryngology, Taizhou People's Hospital, Taizhou, Jiangsu 225300, P.R. China

**Keywords:** laryngeal squamous cell carcinoma, differentially expressed genes, function enrichment, interaction network, clustering analysis

## Abstract

The present study aimed to investigate the differentially expressed genes (DEGs) between laryngeal squamous cell carcinoma (LSCC) samples and non-neoplastic laryngeal squamous cell samples, and the underlying biological mechanism. Gene expression profile data of GSE51985 and GSE10288 were obtained from the Gene Expression Omnibus database. The DEGs between the LSCC and normal samples were identified using the rowtest function in the genefilter package. Hierarchical clustering for DEGs was performed to confirm the distinction between the identified DEGs, and Gene Ontology term and pathway enrichment analyses were performed to determine the underlying function of the DEGs. In addition, protein-protein interaction networks were established to investigate the interactive mechanism of the DEGs. A total of 1,288 upregulated genes and 317 downregulated genes were identified between the LSCC samples and non-neoplastic LSC samples in the GSE51985 dataset, and five upregulated and 26 downregulated genes were identified in the samples from the GSE10288 dataset. The DEGs were clearly distinguished between the LSCC sample and the non-neoplastic LSCC sample by hierarchical clustering. The upregulated genes were predominantly involved in the cell cycle, cell division or focal adhesion, and the 295 upregulated genes formed 374 protein interaction pairs in interaction network analysis. The results revealed that the genes involved in the cell cycle, in cell division or in focal adhesion were associated with the development and progression of LSCC.

## Introduction

Head and neck cancer is the sixth most common type of cancer, with an annual incidence of 700,000 patients worldwide (1,2). It is reported that 20–30% of cases of head and neck cancer are laryngeal tumors (3). Laryngeal squamous cell carcinoma (LSCC), originating in the squamous cells, is the most common type of laryngeal carcinoma, accounting for ~25% of all cases of head and neck squamous cell carcinoma (4), with high mortality rates and a poor prognosis. The five-year survival rates are suggested to be between 52 and 94%, depending on the tumor site, stage and tumor therapy (5,6).

LSCC is considered to be result from the interactions of several genetic and environmental factors (7), including smoking, alcohol consumption (8,9), air pollution and viral infection. Efforts have been made to identify the genes involved in this type of cancer in past few decades. It was demonstrated, by expression profile screening, that protein tyrosine phosphatase receptor type δ is a suppressor gene in LSCC (10). Alterations in the expression of astrocyte elevated gene 1 exerts a predictive value in the prognosis of LSCC (11). Recurrent alterations in the levels of DNA methylation of Fanconi anemia-associated genes including FANCA, BRCA1 and BRCA2 contribute to the development of LSCC (12). However, the pathogenesis of LSCC and associated biological process and pathways remain to be elucidated.

Therefore, the aim of the present study was to determine the pathogenesis of LSCC and to investigate the differences between LSCC and non-neoplastic tissue samples at the molecular level. Differentially expressed genes (DEGs) between LSCC and normal samples were identified, followed by hierarchical clustering and function and pathway enrichment. Furthermore, functional interaction network analysis of the DEGs was performed. The results of the present study may provide novel insights into the therapeutics and assist in improving the survival rate and prognosis of patients with LSCC.

## Materials and methods

### Microarray data

The gene expression profiles of GSE51985 (13) and GSE10288 (14) were obtained from the National Center of Biotechnology Information Gene Expression Omnibus (GEO) database (http://www.ncbi.nlm.gov/geo/). Larynx tissues with regional lymph node metastasis and corresponding adjacent non-neoplastic tissues samples from 10 patients (all males; age range, 52–74 years) who underwent surgery for primary LSCC at the Department of Head and Neck Surgery (Beijing Tongren Hospital, Beijing, China) were available for GSE51985, while GSE10288 contained 13 lesion tissue samples of LSCC from LSCC patients (12 males and 1 female; age range, 44–73 years; two repeats were obtained from each patient) and 10 non-matched normal larynx tissue samples (each had two repeats) from non-neoplastic larynx from the Arnaldo Vieira de Carvalho Hospital (São Paulo, Brazil).

### Data preprocessing

The samples from GSE51985 were annotated using the Illumina HumanHT-12 V4.0 expression beadchip platform (Illumina Inc., San Diego, CA, USA). The expression values were subjected to quantile data normalization using Illumina's Genome Studio v1 software (Illumina Inc.), followed by log_2_ transformation and gene annotation. The microarray detection platform, CAGE Lab-Head and Neck carcinoma cDNA microarray (Department of Biochemistry, Institute of Chemistry, University of São Paulo, Brazil), was used for the annotation of the samples from GSE10288. The probe data were initially normalized by a locally weighted scatterplot smoothing algorithm (http://connection.ebscohost.com/c/articles/28834113/optimized-lowess-normalization-parameter-selection-dna-microarray-data) (15) using GeneSpring software, version 10.0 (Agilent Technologies, Inc., Foster City, CA, USA) and then annotated for gene expression value as described above. In cases where one gene corresponds to multiple probe sets, the average was used as the gene expression value. Subsequently, the normalized values were used to calculate log_2_-transformed Cy5/Cy3 ratios (denoted log_2_-ratios) for each gene using GeneSpring software, version 10.0.

### DEG analysis

To investigate the differences between the LSCC samples and the non-neoplastic LSCC samples, the rowtest algorithm of the genefilter package in R/Bioconductor (www.bioconductor.org/packages/2.3/bioc/html/genefilter.html) (16) was used to identify the DEGs in the two sample groups. The DEGs were required to meet the criteria that |log_2_ fold change (FC)|>1 and P<0.05. Subsequently, the DEGs obtained from the two microarray data were compared using hierarchical clustering in R, version 3.0.2 234 (www.r-project.org). Heatmaps, based on the gene expression values, were produced to verify the distinguished effect of the identified genes on the LSCC and non-neoplastic samples.

### Function and pathway enrichment analysis of DEGs

Gene Ontology (GO) analysis is widely used for functional investigations of large-scale genomic or transcriptomic data (17), which characterizes genes or gene products to a biological process, molecular function and cellular component. Kyoto Encyclopedia of Genes and Genomes (KEGG; www.genome.ad.jp/kegg/kegg2.html pathway analysis is another technique to reveal the biological mechanisms of large numbers of genes derived from high-throughput genomic experiments (18). Database for annotation, visualization and integrated discovery (DAVID; david.abcc.ncifcrf.gov) is one of the most commonly used tools for GO enrichment and pathway analysis (19). As few genes have been sequenced in the GSE10288 profile, only the DEGs of the GSE51985 dataset were subjected to DAVID in the present study, to identify the differences in functions and pathways, with an enrichment significance false discovery rate (FDR) of <0.05. The enrichments of the upregulated and downregulated DGEs were performed separately.

### Functional interaction network analysis of the upregulated DEGs

To gain further insights into the functional coordination of the DEGs, the human protein reference database (HPRD; www.hprd.org) was used to examine the interacting pairs associated with the upregulated DEGs. The interacting pairs were visualized via Cytoscape (www.cytoscape. org) (20). Additionally, the significant pathways associated with these pairs were enriched via DAVID, with the threshold as FDR<0.05.

## Results

### DEG screening and comparison

**Figure 1:**
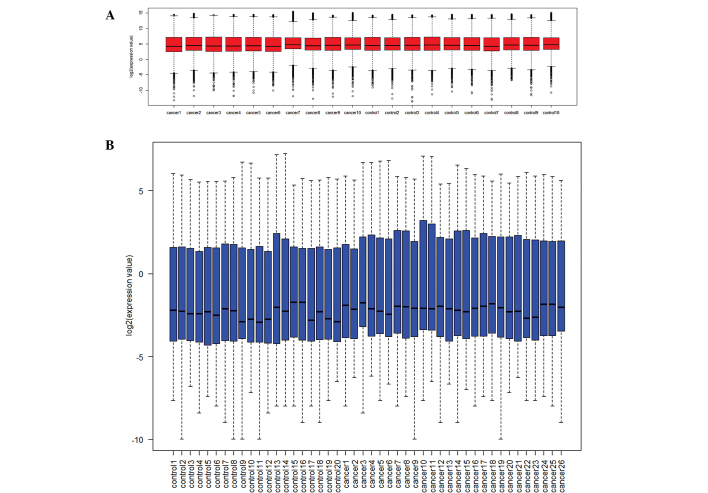
Gene expression value cassette of (A) GSE51985 and (B) GSE10288 after normalization. The horizontal axis represents the samples and the vertical axis represents the gene expression values of log_2_ transformation. Black lines within the boxes indicate the median. The above and the below bars indicate 1/4 and 3/4 of the gene expression value.

Following normalization of the microarray data (Fig. 1), a rowtest algorithm was used to identify the DEGs between the LSCC samples and the non-neoplastic LSC samples. A total of 1,605 genes were identified as significantly differentially expressed in the samples from the GSE51985 dataset, among which 1,288 genes were upregulated and 317 genes were downregulated in the LSCC samples, compared with the non-neoplastic LSC samples. Similarly, 31 genes were identified as DEGs in the samples from the GSE10288 dataset, including five upregulated and 26 downregulated genes (Table I). Following comparisons of the DEGs in the GSE51985 and GSE10288 datasets, four genes were found to be differentially expressed in the two datasets. These genes were dynein, axonemal, heavy Chain 1 (*DNAH1*), ubiquitin C (*UBC*), early endosome antigen 1 (*EEA1*) and ubiquitin specific peptidase (*EEA1*), of which, *DNAAH1* was downregulated in the LSCC sample from the two datasets, and the other three DEGs exhibited a different trend of expression in the two datasets, which may have been attributed to the different sources of the samples for the two datasets.

### DEG clustering analysis

**Figure 2:**
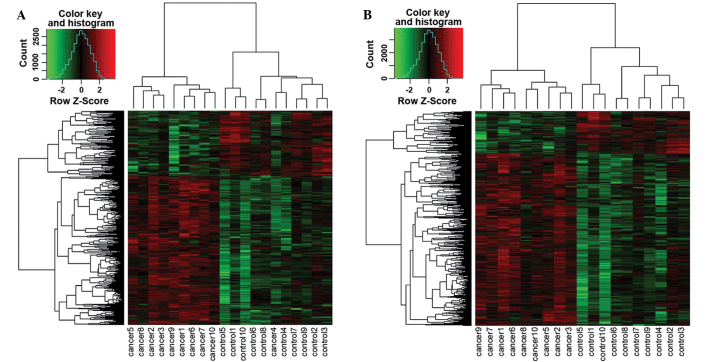
Hierarchical clustering heatmap for differentially expressed genes between laryngeal squamous cell carcinoma samples and non-neoplastic samples. (A) The heatmap prior to removal of the cancer sample 4; (B) the heatmap subsequent to the removal of cancer sample 4. The heatmap was constructed using Euclidean distance with average linkage. The Z-score centered log_2_-transformed gene in each sample is presented using a color scale. The gene expression differences are highlighted in green (downregulation) and red (upregulation).

The heatmap of the DEG hierarchical clustering (Fig. 2A) demonstrated that the cancer 4 sample in LSCC was clustered into the non-neoplastic LSCC sample cluster, and no significant difference was observed in the DEGs between the cancer 4 sample and the corresponding control 4 sample. Therefore, the cancer 4 sample was excluded from the subsequent hierarchical clustering. As expected, the selected DEGs were well distinguished between the LSCC sample and the non-neoplastic LSCC sample (Fig. 2B).

### GO enrichment analysis and pathway analysis

The results of the GO enrichment analysis are shown in Table II, which demonstrated that the upregulated genes were predominantly involved in the cell cycle and cell division processes (34 GO terms). The downregulated genes included only a few genes, which were involved in the oxidation reduction process. The pathway enrichment analysis revealed that no upregulated or downregulated DEGs were enriched in specific pathways.

### Functional network analysis of the upregulated DEGs

**Figure 3:**
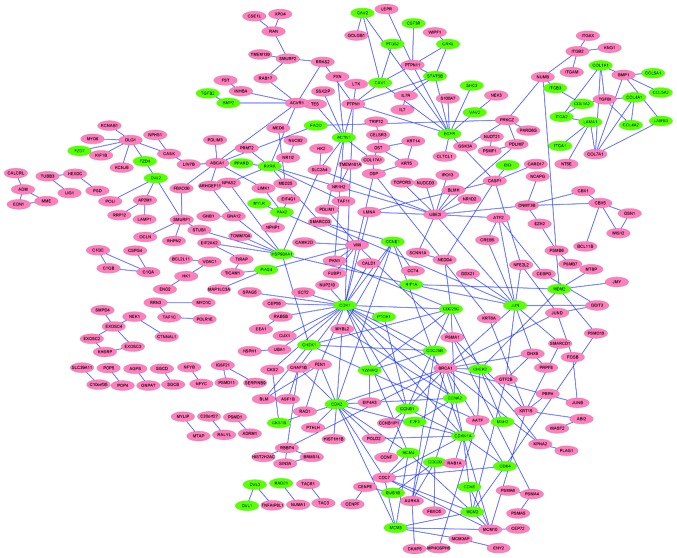
Functional network of the upregulated genes in laryngeal squamous cell carcinoma samples. Pink nodes indicate the upregulated genes. Green nodes indicate genes significantly enriched in cell cycle, normal cancer, small cell lung cancer and adhesion plaque pathways. Black lines indicate associated pairs among the network.

HPRD consists of 39,240 protein pairs and 10,200 proteins. The LSCC upregulated genes were mapped to HPRD, and 374 interacting pairs, including 294 upregulated DEGs, were identified and used for construction of the interaction network (Fig. 3). Among the 294 genes, 64 were enriched in several specific pathways (Table III), including the cell cycle (hsa04110), pathways in cancer (hsa05200), small cell lung cancer (has05222) and focal adhesion (hsa04510). Overall, there were 21 genes enriched in the focal adhesion pathway, including epidermal growth receptor (*EGFR*), caveolin 2 (*CAV2*), collagen type V, alpha 1 (*COL5A1*) and laminin alpha 1 (*LAMA1*).

## Discussion

Gene expression levels in disease reveal the potential biological mechanism of the disease. The present study downloaded two datasets of gene expression profiles from GEO. A total of 1,605 genes were identified as significantly differentially expressed in samples from the GSE51985 dataset and 31 genes were identified as DEGs in samples from the GSE10288 dataset. Although identified in different samples, certain genes were revealed to be differentially expressed in the two profiles, including *DNAH1.*

*DNAH1*, which codes the proteins of the axonemal dynein cluster, is a large subunit of dynactin. The *DNAH1* mutation has been detected in exome-sequenced colorectal cancer and melanoma specimens (21). *DNAH1* is involved in the significant differences in DNA copy number between adenocarcinoma and squamous cell carcinoma (22). Furthermore, *DNAH1* is putatively involved in cell motility and migration (23). Cancer cells move within tissues during invasion and metastasis through their own motility (24), and the migratory mechanisms can respond to different conditions (25). Multiple genes associated with cell motility are reported to be deregulated in human cancer (26). *DNAH1* was also downregulated in the LSCC samples used in the present study, therefore, it was suggested that *DNAH1* may exert its effect in LSCC through its involvement in cell motility.

By analyzing two datasets of LSCC samples, the present study revealed that the DEGs and their function in the LSCC sample demonstrated similar characteristics with general types of cancer, particularly the upregulated genes, as they were significantly involved in cell cycle, likely to increase cell proliferation rate and lead to tumorigenesis. *CDC7* and *CDK1* were among the genes enriched in the cell cycle pathway. CDKs are threonine/serine protein kinases, the activities of which depend on the action and binding of cyclin partners (27). Tumor-associated cell cycle defects are usually mediated by alteration in the activity of CDK (28). As a key regulator of the cell cycle, *CDK1* is a powerful therapeutic target for cancer inhibitors (29). In precursor lesions and esophageal adenocarcinoma, the expression of *CDC72*/*CDK1* serves as a diagnostic and cancer progression marker (30).

In addition, the present study demonstrated that certain genes were also involved in the LSCC bifocal adhesion pathway, including *EGFR*. Focal adhesion kinase (FAK) is involved in cancer cell tumor formation and progression (31). Lymph node metastasis in esophageal SCC is associated with the overexpression of FAK (32), which is also observed in head and neck squamous cell carcinoma (33). The simultaneous inhibition of EGFR and the FAK pathway increases the apoptotic response of cancer cells (34). In addition, in colon and breast cancer cells, FAK survival signaling exerts its roles by combining with EGFR (35). EGFR-targeted therapy is used in the treatment of head and neck cancer via targeting the pathways involved in tumor growth, angiogenesis, metastasis and invasion (36), for example, the EGFR inhibitor, gefitinib, has been used in clinical practice in the treatment of head and neck squamous cell carcinoma (37). High levels of EGFR can indicate patients with laryngeal cancer with a poor prognosis (38). Therefore, the present study hypothesized that EGFR is involved in the development of LSCC via the FAK pathway. Furthermore, genes of the integrin family are also involved in LSCC via the FAK pathway, including integrin α1 (*ITGA1*) and integrin β3 (*ITGB3*). It is reported that the combination of *ITGB3* with *SDC4* may result in the activation of FAK (39). Integrin/FAK signaling can control tumor initiation, growth and progression into malignant squamous cell carcinoma (40).

In conclusion, with the assistance of high-throughput microarray data analysis, based on bioinformatics methods, the present study identified several DEGs, as well as their abnormal functions and pathways, in LSCC. The associations identified between the DEGs and their relative biological processes offer novel insights into the mechanism underlying LSCC.

## Figures and Tables

**Table I:** DEGs in the GSE51985 and GSE10288 datasets.

Dataset	Total genes	Upregulated genes	Downregulated genes	DEGs
GSE10288	134	5	26	31
GSE51985	21410	1288	317	1605

DEGs, differentially expressed genes.

**Table II:** GO enrichment analysis of differentially expressed genes.

GO term	Number	Fold enrichment	FDR
Downregulated gene			
GO:0055114 oxidation reduction	25	2.846	0.011
Upregulated gene			
GO:0000278 mitotic cell cycle	71	2.937	6.000^−13^
GO:0022402 cell cycle process	91	2.465	2.220^−12^
GO:0007049 cell cycle	108	2.130	9.210^−11^
GO:0022403 cell cycle phase	67	2.477	1.650^−08^
GO:0051301 cell division	50	2.594	2.070^−06^
GO:0000087 M phase of mitotic cell cycle	42	2.869	2.570^−06^
GO:0000280 nuclear division	41	2.852	4.990^−06^
GO:0007067 mitosis	41	2.852	4.990^−06^
GO:0000279 M phase	52	2.419	1.100^−05^
GO:0048285 organelle fission	41	2.740	1.650^−05^
GO:0031396 regulation of protein ubiquitination	24	3.673	1.500^−04^
GO:0006260 DNA replication	33	2.658	0.001
GO:0051726 regulation of cell cycle	47	2.173	0.002
GO:0034621 cellular macromolecular complex subunit organization	49	2.100	0.002
GO:0031398 positive regulation of protein ubiquitination	20	3.644	0.003
GO:0007398 ectoderm development	33	2.538	0.003
GO:0010604 positive regulation of macromolecule metabolic process	92	1.643	0.004
GO:0008544 epidermis development	31	2.578	0.005
GO:0051340 regulation of ligase activity	19	3.590	0.006
GO:0051325 interphase	22	3.176	0.007
GO:0065004 protein-DNA complex assembly	20	3.363	0.009
GO:0033554 cellular response to stress	66	1.784	0.010
GO:0031400 negative regulation of protein modification process	23	2.958	0.014
GO:0043161 proteasomal ubiquitin-dependent protein catabolic process	21	3.151	0.014
GO:0010498 proteasomal protein catabolic process	21	3.151	0.014
GO:0051438 regulation of ubiquitin-protein ligase activity	18	3.532	0.016
GO:0051329 interphase of mitotic cell cycle	21	3.120	0.017
GO:0034622 cellular macromolecular complex assembly	43	2.069	0.017
GO:0051439 regulation of ubiquitin-protein ligase activity during mitotic cell cycle	17	3.664	0.018
GO:0031145 anaphase-promoting complex-dependent proteasomal ubiquitin-dependent protein catabolic process	16	3.767	0.026
GO:0051351 positive regulation of ligase activity	17	3.564	0.027
GO:0051247 positive regulation of protein metabolic process	35	2.204	0.036
GO:0032270 positive regulation of cellular protein metabolic process	34	2.233	0.037
GO:0051437 positive regulation of ubiquitin-protein ligase activity during mitotic cell cycle	16	3.601	0.045

GO, Gene Ontology; FDR, false discovery rate.

**Table III:** KEGG pathway enrichment results for the differentially expressed genes in the functional interaction network.

KEGG pathway	Count	Fold enrichment	FDR	Genes
hsa04110:Cell cycle	23	5.536331361	6.87E-08	CDC7, CDK1, E2F3, CDK6, CHEK1, CDC20, MCM2, CHEK2, CDK4, CDC25C, MCM3, MCM4, CDK2, TGFB2, CDC25B, CCNB1, CCNE1, CDKN1A, RAD21, YWHAQ, BUB1B, MDM2, CCNA2
hsa05200:Pathways in cancer	35	3.210690576	1.18E-06	BID, CKS1B, E2F3, PPARD, PTGS2, STAT5B, TGFB2, CCNE1, LAMB3, CSF3R, DVL2, EGFR, DVL3, BMP2, COL4A2, HSP90AA1, COL4A1, MSH2, RXRA, ITGA2, CDK6, FADD, CDK4, FZD4, CDK2, FZD7, DVL1, LAMA1, CDKN1A, CRKL, HIF1A, PIAS4, JUN, MDM2, PTCH1
hsa05222:Small cell lung cancer	14	5.014792899	0.003233743	CKS1B, COL4A2, E2F3, COL4A1, PTGS2, RXRA, ITGA2, CDK6, CDK4, CDK2, CCNE1, LAMA1, LAMB3, PIAS4
hsa04510:Focal adhesion	21	3.143601519	0.008951954	EGFR, CAV2, CAV1, COL4A2, COL4A1, ITGA1, ITGA2, ACTN1, ITGB3, VAV2, COL5A2, COL5A1, LAMA1, LAMB3, CRKL, PAK2, JUN, COL1A2, COL1A1, SHC3, MYLK

FDR, false discovery rate; KEGG, Kyoto Encyclopedia of Genes and Genomes.

## References

[1] Jemal A, Bray F, Center MM, Ferlay J, Ward E, Forman D (2011). Global cancer statistics. CA Cancer J Clin.

[2] Gleeson M, Browning G, Burton Martin J (2008). Scott-Brown's Otorhinolaryngology. Head and Neck Surgery.

[3] Schrijvers M (2011). New prognostic markers to predict clinical outcome in patients with laryngeal cancer. Radiother Oncol.

[4] Hunter KD, Parkinson EK, Harrison PR (2005). Profiling early head and neck cancer. Nat Rev Cancer.

[5] Dequanter D, Lothaire P (2008). The role of salvage surgery in organ preservation strategies in advanced head and neck cancer. B-ENT.

[6] León X, López M, García J, Viza I, Orús C, Quer M (2007). Supracricoid laryngectomy as salvage surgery after failure of radiation therapy. Eur Arch Otorhinolaryngol.

[7] Liu M, Wu H, Liu T (2009). Regulation of the cell cycle gene, BTG2, by miR-21 in human laryngeal carcinoma. Cell Res.

[8] Sasaki CT, Jassin B (2001). Cancer of the pharynx and larynx. Am J Med.

[9] Manjarrez ME, Ocadiz R, Valle L (2006). Detection of human papillomavirus and relevant tumor suppressors and oncoproteins in laryngeal tumors. Clin Cancer Res.

[10] Giefing M, Zemke N, Brauze D (2011). High resolution ArrayCGH and expression profiling identifies PTPRD and PCDH17/PCH68 as tumor suppressor gene candidates in laryngeal squamous cell carcinoma. Genes Chromosomes Cancer.

[11] Liu Y, Li G, Su ZW (2013). Expression of astrocyte elevated gene-1 protein and its clinical significance in laryngeal squamous cell carcinoma. Zhonghua Bing Li Xue Za zhi.

[12] Szaumkessel M, Richter J, Giefing M (2011). Pyrosequencing-based DNA methylation profiling of Fanconi anemia/BRCA pathway genes in laryngeal squamous cell carcinoma. Int J Oncol.

[13] Lian M, Fang J, Han D (2013). Microarray gene expression analysis of tumorigenesis and regional lymph node metastasis in laryngeal squamous cell carcinoma. PLoS One.

[14] Colombo J, Fachel AA, De Freitas Calmon M, Cury PM, Fukuyama EE, Tajara EH, Cordeiro JA, Verjovski-Almeida S, Reis EM, Rahal P (2009). Gene expression profiling reveals molecular marker candidates of laryngeal squamous cell carcinoma. Oncol Rep.

[15] Berger JA, Hautaniemi S, Järvinen AK, Edgren H, Mitra SK, Astola J (2004). Optimized LOWESS normalization parameter selection for DNA microarray data. BMC Bioinformatics.

[16] Tilford CA, Siemers NO (2009). Gene set enrichment analysis. Methods Mol Biol.

[17] Hulsegge I, Kommadath A, Smits MA (2009). Globaltest and GOEAST: two different approaches for gene ontology analysis. BMC Proc.

[18] Kanehisa M, Goto S (2000). KEGG: Kyoto encyclopedia of genes and genomes. Nucleic Acids Res.

[19] Huang da W, Sherman BT, Lempicki RA (2009). Systematic and integrative analysis of large gene lists using DAVID bioinformatics resources. Nat Protoc.

[20] Shannon P, Markiel A, Ozier O (2003). Cytoscape: a software environment for integrated models of biomolecular interaction networks. Genome Res.

[21] McNerney ME, Brown CD, Peterson AL (2014). The spectrum of somatic mutations in high-risk acute myeloid leukaemia with-7/del (7q). Br J Haematol.

[22] Son JW, Jeong KJ, Jean WS (2011). Genome-wide combination profiling of DNA copy number and methylation for deciphering biomarkers in non-small cell lung cancer patients. Cancer Lett.

[23] Milosevic J, Klinge J, Borg AL, Foukakis T, Bergh J, Tobin NP (2013). Clinical instability of breast cancer markers is reflected in long-term in vitro estrogen deprivation studies. BMC Cancer.

[24] Yamazaki D, Kurisu S, Takenawa T (2005). Regulation of cancer cell motility through actin reorganization. Cancer Sci.

[25] Friedl P, Wolf K (2003). Tumour-cell invasion and migration: diversity and escape mechanisms. Nat Rev Cancer.

[26] Sahai E (2005). Mechanisms of cancer cell invasion. Curr Opin Genet Dev.

[27] Malumbres M, Pevarello P, Barbacid M, Bischoff JR (2008). CDK inhibitors in cancer therapy: what is next?. Trends Pharm Sci.

[28] Malumbres M, Barbacid M (2009). Cell cycle, CDKs and cancer: a changing paradigm. Nat Rev Cancer.

[29] Wang Q, Su L, Liu N, Zhang L, Xu W, Fang H (2011). Cyclin dependent kinase 1 inhibitors: a review of recent progress. Curr Med Chem.

[30] Hansel DE, Dhara S, Huang RC (2005). CDC2/CDK1 expression in esophageal adenocarcinoma and precursor lesions serves as a diagnostic and cancer progression marker and potential novel drug target. Am J Surg Pathol.

[31] McLean GW, Carragher NO, Avizienyte E, Evans J, Brunton VG, Frame MC (2005). The role of focal-adhesion kinase in cancer–a new therapeutic opportunity. Nat Rev Cancer.

[32] Miyazaki T, Kato H, Nakajima M (2003). FAK overexpression is correlated with tumour invasiveness and lymph node metastasis in oesophageal squamous cell carcinoma. Br J Cancer.

[33] Canel M, Secades P, Rodrigo JP (2006). Overexpression of focal adhesion kinase in head and neck squamous cell carcinoma is independent of fak gene copy number. Clin Cancer Res.

[34] Golubovskaya VM, Gross S, Kaur AS (2003). Simultaneous inhibition of focal adhesion kinase and SRC enhances detachment and apoptosis in colon cancer cell lines. Mol Cancer Res.

[35] Golubovskaya VM, Cance W (2010). Focal adhesion kinase and p53 signal transduction pathways in cancer. Front Biosci (Landmark Ed).

[36] Chen LF, Cohen EE, Grandis JR (2010). New strategies in head and neck cancer: understanding resistance to epidermal growth factor receptor inhibitors. Clin Cancer Res.

[37] Erjala K, Sundvall M, Junttila TT (2006). Signaling via ErbB2 and ErbB3 associates with resistance and epidermal growth factor receptor (EGFR) amplification with sensitivity to EGFR inhibitor gefitinib in head and neck squamous cell carcinoma cells. Clin Cancer Res.

[38] Maurizi M, Almadori G, Ferrandina G (1996). Prognostic significance of epidermal growth factor receptor in laryngeal squamous cell carcinoma. Br J Cancer.

[39] Erdem M, Erdem S, Sanli O (2014). Up-regulation of TGM2 with ITGB1 and SDC4 is important in the development and metastasis of renal cell carcinoma. Urol Oncol.

[40] Schober M, Fuchs E (2011). Tumor-initiating stem cells of squamous cell carcinomas and their control by TGF-β and integrin/focal adhesion kinase (FAK) signaling. Proc Natl Acad Sci USA.

